# Physiological and molecular mechanisms of *Acacia melanoxylon* stem in response to boron deficiency

**DOI:** 10.3389/fpls.2023.1268835

**Published:** 2023-10-27

**Authors:** Zhaoli Chen, Xiaogang Bai, Bingshan Zeng, Chunjie Fan, Xiangyang Li, Bing Hu

**Affiliations:** ^1^ Key Laboratory of State Forestry and Grassland Administration on Tropical Forestry, Research Institute of Tropical Forestry, Chinese Academy of Forestry, Guangzhou, Guangdong, China; ^2^ College of Agriculture and Biology, Zhongkai University of Agriculture and Engineering, Guangzhou, Guangdong, China

**Keywords:** boron deficiency, transcriptome, cell wall, hormone, *Acacia melanoxylon*

## Abstract

Boron is an essential micronutrient for plant growth as it participates in cell wall integrity. The growth and development of *Acacia melanoxylon* stem can be adversely affected by a lack of boron. To explore the mechanism of boron deficiency in *A. melanoxylon* stem, the changes in morphological attributes, physiological, endogenous hormone levels, and the cell structure and component contents were examined. In addition, the molecular mechanism of shortened internodes resulting from boron deficiency was elucidated through transcriptome analysis. The results showed that boron deficiency resulted in decreased height, shortened internodes, and reduced root length and surface area, corresponding with decreased boron content in the roots, stems, and leaves of *A. melanoxylon*. In shortened internodes of stems, oxidative damage, and disordered hormone homeostasis were induced, the cell wall was thickened, hemicellulose and water-soluble pectin contents decreased, while the cellulose content increased under boron deficiency. Furthermore, plenty of genes associated with cell wall metabolism and structural components, including *GAUTs*, *CESAs*, *IRXs*, *EXPs*, *TBLs*, and *XTHs* were downregulated under boron deficiency. Alterations of gene expression in hormone signaling pathways comprising IAA, GA, CTK, ET, ABA, and JA were observed under boron deficiency. TFs, homologous to *HD1*s, *NAC10*, *NAC73*, *MYB46s*, *MYB58*, and *ERF92s* were found to interact with genes related to cell wall metabolism, and the structural components were identified. We established a regulatory mechanism network of boron deficiency-induced shortened internodes in *A. melanoxylon* based on the above results. This research provides a theoretical basis for understanding the response mechanism of woody plants to boron deficiency.

## 1 Introduction

Boron is an essential trace mineral element for plants (Warington, 1923) and plays a critical role in plasma membrane integrity and function, cell wall structure and function, carbohydrate and nucleic acid metabolism, phenol, and hormone metabolism, as well as respiration and photosynthesis (García-Sánchez et al., 2020; Bolaños et al., 2023; Chen et al., 2023). Boron primarily exists in the form of boric acid (H_3_BO_3_) in soil, which is prone to leaching during heavy rainfall (Shorrocks, 1997; Brdar-Jokanović, 2020). In more than 80 countries globally, boron deficiency has become a widespread concern problem in both agriculture and forestry. Boron deficiency plants exhibit diverse visible symptoms in vegetative and reproductive organs, such as reduced root growth, suppressed plant height, decreased leaf area, lost apical shoot dominance, and reduced fertility (Shorrocks, 1997; Wang et al., 2015).

Boron is mainly involved in the formation and structural integrity of the primary cell wall by crosslinking pectin polysaccharide rhamnogalacturonan II (RG-II) (O'Neill et al., 2001; Camacho-Cristóbal et al., 2011). And nearly 90% of cellular boron is positioned on the cell wall (Kobayashi et al., 1997). When boron is lacking, the cell wall structure is disorganized, and the intercellular pectin polysaccharides are impacted, thereby preventing cell wall integrity (Martín-Rejano et al., 2011). When cell wall integrity is impaired, multiple signaling pathways are induced, such as hormone signaling, reactive oxygen species (ROS) accumulation, and the production of other cell wall components (Vaahtera et al., 2019). In *Arabidopsis* seedlings, the impaired cell wall integrity caused by boron deficiency triggers ethylene (ET), auxin (IAA), and ROS signals, consequently resulting in the reduction of root cell elongation (Camacho-Cristóbal et al., 2015). Unfavorable boron conditions also result in lipid peroxidation and imbalanced antioxidant enzyme activities through the excessive buildup of oxidative stress (Tewari et al., 2010). Specifically, boron deficiency increases the content of malondialdehyde (MDA) and proline (Pro), as well as upregulates the activity of lipoxygenase (LOX), and then regulates the activity of antioxidative enzymes, such as superoxide dismutase (SOD), catalase (CAT), and peroxidase (POD) (Molassiotis et al., 2006; Tewari et al., 2010; Yin et al., 2022).

Although the molecular mechanism of plant response to boron deficiency is not well studied, some results have been found at the transcriptional level. In the case of *Neolamarckia cadamba*, Yin et al. (2022) observed that the phenylalanine ammonia-lyase and phenylpropanoid biosynthesis pathways were induced under boron deficiency, resulting in increased shoot tip lignification. The expression levels of the genes related to the synthesis of pectin and cellulose in *N. cadamba* mature leaves were altered in response to boron deficiency stress. Moreover, numerous transcription factors (TFs) also serve as the switches of the regulatory signal cascade in response to the boron deficiency stress process. The earliest reported TFs involved in boron stress responses is *AtWRKY6* in *Arabidopsis* (Kasajima et al., 2010). A recent study further noted that *BnaWRKYs* participated in the response to low boron, and *BnaA9.WRKY47* contributed to the adaptation of *Brassica napus* to boron deficiency through upregulating *BnaA3.NIP5;1* (a boron transporter gene) expression to facilitate efficient boron uptake (Feng et al., 2020). The study conducted by Song et al. (2021) utilized transcriptome analysis to find numerous differentially expressed genes (DEGs) related to antioxidant enzymes, TFs, and boron transporters, which revealed the response mechanism of boron deficiency tolerance in leaves of *Beta vulgaris* seedlings.

*Acacia melanoxylon* is an evergreen and fast-growing tree belonging to the Leguminosae family and Mimosaceae subfamily (Bradbury et al., 2011). Due to its strong adaptability, good material properties, and short rotation period, it is an ideal species that integrates economic, ecological, and greening functions. *A. melanoxylon* is commonly distributed in areas with high rainfall and slightly acidic soil, such as Australia, South China, Brazil, and Ethiopia (Searle, 2000). This tree species is easily affected by a lack of boron which hinders its growth. Since the development of stem is a significant economic indicator for forest tree species, the present study explored the morphological and physiological effects of boron deficiency on *A. melanoxylon* stem and used RNA-seq technology to identify the DEGs related to the internode shortening caused by the deficiency. Furthermore, it revealed the interaction network among the cell wall organization or biogenesis, hormone signal transduction pathways, and TFs in response to boron deficiency stress, improving the understanding of the stem response mechanism to boron deficiency in tree species.

## 2 Materials and methods

### 2.1 Plant materials and culture conditions

The elite *A. melanoxylon* clone SR17 was selected as plant material. And two-month-old shoots were cultured into plastic containers (30 cm × 26 cm × 14 cm) with 1/2 MS (Murashige & Skoog) nutrient solution supplemented with the following concentrations of macro- and micro-nutrients; 10.31 mM NH_4_NO_3_, 9.39 mM KNO_3_, 1.50 mM CaCl_2_, 0.75 mM MgSO_4_, 0.63 mM KH_2_PO_4_, 0.06 μM CoCl_2_, 0.05 μM CuSO_4_·5H_2_O, 50 μM FeNaEDTA, 50 μM MnSO_4_·H_2_O, 0.52 μM Na_2_MoO_4_·2H_2_O, 2.5 μM KI, 14.96 μM ZnSO_4_·7H_2_O. Then, the plants with consistent plant height and growth state were selected for further boron deficiency treatment (without H_3_BO_3_). The control was cultured with the nutrient solution with 50 μM of H_3_BO_3_. Each treatment was performed in three replications with 16 plants in each replication. The fresh nutrient solution was replaced every three days, and the pH was maintained at 5.5-6.0 to balance ion absorption and distribution. Experiments were conducted in a greenhouse facility under natural sunlight conditions, 75% atmospheric humidity, and a temperature range of 23-28 °C.

### 2.2 Growth parameters measurement and sample collection

After 60 days under boron deficiency, plant height, branch number, and internode length of the apical section (1st, 2nd, 3rd) were measured. Meanwhile, the total root length, root diameter, and root surface area were calculated by using a scanner and root image analysis software WinRHIZO Pro (Regent Instruments, QC, Canada).

Roots, stems, and leaves were also harvested and immediately frozen in liquid nitrogen and then transferred to a -80 °C refrigerator for further physiology, hormones, and RNA-seq analysis. The 2.5 g fresh samples were dried in an oven at 75 °C for a constant weight and then determined for the boron content.

### 2.3 Boron content measurement

0.5 g dried samples were ground to a fine powder and underwent ashing at 500 °C. Then, the ash was digested in 0.1 M HCl for 30 min and filtered using quantitative filter paper. Finally, the boron content in plants was measured using the curcumin colorimetry method with a UA-spectrophotometer (UV-2450, Shimadzu, Kyoto, Japan) (Dible et al., 1954). The calculation formulae are as follows:


$$\begin{array}{c} Boron\;accumulation\;(\mu g\cdot plant^{-1})\\ \;=\;boron\;content\;(\mu g\cdot g^{-1})\;\times \;corresponding\;dry\;weight\;(g\cdot plant^{-1}) \end{array}$$

$$\begin{array}{c} Boron\;transport\;coefficient\;(BTC)\\ \;=\;shoot\;boron\;content\;(\mu g\cdot plant^{-1})/root\;boron\;content\;(\mu g\cdot plant^{-1}) \end{array}$$

$$\begin{array}{c} Boron\;efficiency\;coefficient\;(BEC)\\ =\;total\;dry\;weight\;of\;B0(g)/total\;dry\;weight\;of\;control(g) \end{array}$$

### 2.4 Physiological indicators measurement

The frozen stems (0.10 g) were selected for physiological analysis. MDA content was measured using the 20% (*w/v*) trichloroacetic (TCA) and 0.5% (*w/v*) thiobarbituric acid (TBA) method (Heath and Packer, 1968). LOX activity was determined by analyzing LOX-catalyzed linoleic acid oxidation at 234 nm (Pérez et al., 1999). Pro content was extracted from 3% (*w/v*) aqueous sulfosalicylic acid and estimated by ninhydrin reagent at 520 nm (Bates et al., 1973). SOD activity was evaluated using the nitrotetrazolium blue chloride (NBT) photochemical reduction method (Giannopolitis and Ries, 1977). POD activity was measured based on guaiacol oxidation at 470 nm using hydrogen peroxide (H_2_O_2_). CAT activity was determined by measuring the disappearance of H_2_O_2_ at 240 nm (Rao et al., 1996). Each assay had three independent replications.

### 2.5 Endogenous hormone contents measurement

An improved double antibody sandwich enzyme-linked immunosorbent assay (ELISA) was used to quantify IAA, CTK, GA, ABA, ET, and JA in stems according to the kit’s instructions (Shanghai Enzyme-linked Biotechnology, Shanghai, China). Specifically, the frozen stems (0.20 g) were ground in liquid nitrogen and homogenized in 2 ml phosphate-buffered saline (PBS; 0.01 M, pH 7.5). After centrifugation at 10000 xg for 10 min, the supernatant (50.00 μL) and biotinylated plant corresponding antibodies were added to wells and incubated at 37°C for 30 min. The liquid was removed, and the plates were washed five times with washing buffer. Enzyme conjugate liquid (50 µL) was added to wells and incubated at 37 °C for 30 min, and the plates were washed five times with washing buffer. Next, color reagent A (50 µL) and B (50 µL) were added to wells and incubated at 37°C for 10 min. Finally, the reaction was terminated by adding color reagent C (50 µL). A standard curve was generated using five known contents of hormones, the absorbance (OD value) was measured at 450 nm, and the regression equation of the standard curve was used to determine the content of each hormone. Each assay had three independent replications.

### 2.6 Transmission electron microscope analysis

The TEM slices were performed using the method of Kong et al. (2013) with slight modifications. Specifically, stems from the same parts under different boron treatments were cut into small pieces (1 mm × 1 mm). The samples were fixed in glutaraldehyde in phosphate buffer solution (PBS, 0.1 M) for 12 h at 4°C. Then, the tissue blocks were rinsed four times using 0.1 M PBS (pH 7.4) and post-fixed for 2-3 h with 1% buffered osmium tetroxide, followed by rinsing in 0.1 M PBS (pH 7.4) for three times. Next, the samples were dehydrated using an increasing ethanol concentration series (30, 50, 70, 80, 95, 100, 100) and transferred into a mixture of ethanol and acetone (3:1, 1:1, 1:3, 0:1). Ultrathin sections were stained with 2% uranyl acetate and lead citrate, and were examined with a TEM (Hitachi, HT7800/HT7700, Japan). The cell wall thickness was measured (20 replicates for one treatment) with Image J’s scale tool based on the TEM image scale.

### 2.7 Cellulose, hemicellulose, and water-soluble pectin content measurement

The contents of cellulose and hemicellulose of stems were determined as follows: 3.0 g dried samples were ground to a fine powder and digested in a mixture of acidic detergent and 1-octanol for 60 minutes. Then, the digestion liquor was filtered with a funnel. The residue was digested in H_2_SO_4_ for 3 hours, then filtered again and washed with hot water until neutral. Next, the residue was dried to constant weight at 105°C. Finally, the cellulose and hemicellulose content was determined using the gravimetric technique in a fully automated fiber analysis system (Fibertech TM 8000, FOSS, Denmark). Each assay had three independent replications.

0.3 g fresh stems were ground into powder and quickly homogenized in 1 mL 80% ethanol. The samples were incubated in a 95°C water bath for 20 min. After centrifugation at 4000 xg for 10 min, the precipitate was collected. The final residue was defined as a crude cell wall after being washed with 1.5 mL 80% ethanol and acetone. 3 mg dried crude cell wall dissolved in 1 mL anhydrous sodium acetate (pH 6.5) was shaken for 15 hours. After centrifugation at 8000 xg for 10 min, the supernatant was collected. The content of WSP was measured by reading the absorbance at 530 nm with a tube photometer. Each assay had three independent replications.

### 2.8 Transcriptomics analysis

The RNA of stems was extracted using the RNAprep Pure Plant Kit (DP441, Tiangen, China). RNA quality and quantity were verified with an RNA integrity number (RIN) greater than 7.2, 260/280 ratio of 1.8 to 2.0, 260/230 ratio of 1.8 to 2.2, and a concentration greater than 300 ng·µL^-1^, RNA samples were selected for further experiments. First-strand cDNA was synthesized using the SuperScriptTM II Reverse Transcriptase kit (18-064-022, Invitrogen, USA).

RNA-Seq of qualified libraries was performed using the Illumina HiSeq4000 platform, with a sequencing strategy of 150 bp paired-end. The clean reads were mapped to the *A. melanoxylon* reference genome sequence (unpublished) using HISAT2 (Kim et al., 2015). After alignment, mRNA expression levels were calculated by combining RNA-Seq by Expectation Maximization (RSEM) with Fragments Per Kilobase of exon model per Million mapped fragments (FPKM) values (Trapnell et al., 2010; Li and Dewey, 2011). DEGs were identified using DESeq2 with |Log_2_(fold-change) | ≥ 1, FDR ≤ 0.05, and P-value< 0.05 (Love et al., 2014). All DEGs were mapped to GO terms in the GO database and pathways in the KEGG database. The TBtools software was used to delineate heatmaps based on the DEG results (Chen et al., 2020). The protein interaction networks were visualized with Cytoscape (Shannon et al., 2003). In addition, the RNA-seq data were submitted to NCBI with the submission number: PRJNA995919.

### 2.9 qRT-PCR validation and expression analysis

For quantitative real-time PCR (qRT-PCR), the same RNA and cDNA stem samples used for transcriptome sequencing were utilized. The qRT-PCR test was performed using the TB Green Premix Ex TaqTM kit (RR820, TaKaRa, China). The qRT-PCR reaction system and procedures were carried out according to the kit requirements. All qRT-PCR amplifications were repeated three times. All the genes were normalized against the level of protein phosphatase type 2A (*evm.TU.Chr3.536 PP2a*). The details of the gene-specific primers are listed in **Supplementary File 1**.

### 2.10 Statistical analysis

SPSS 26.0 (SPSS Inc., Chicago, USA) software was employed to conduct variance analysis. The minimum significant difference method (LSD) at P-value< 0.05 and 0.01. Principal component analysis (PCA), Pearson correlation analysis, and figures were prepared using Origin 2021 (OriginLab Co., Massachusetts, USA). Significant and extremely significant differences were expressed by * and **, respectively.

## 3 Results

### 3.1 Effect of boron deficiency on plant morphological attributes

**Figure 1:**
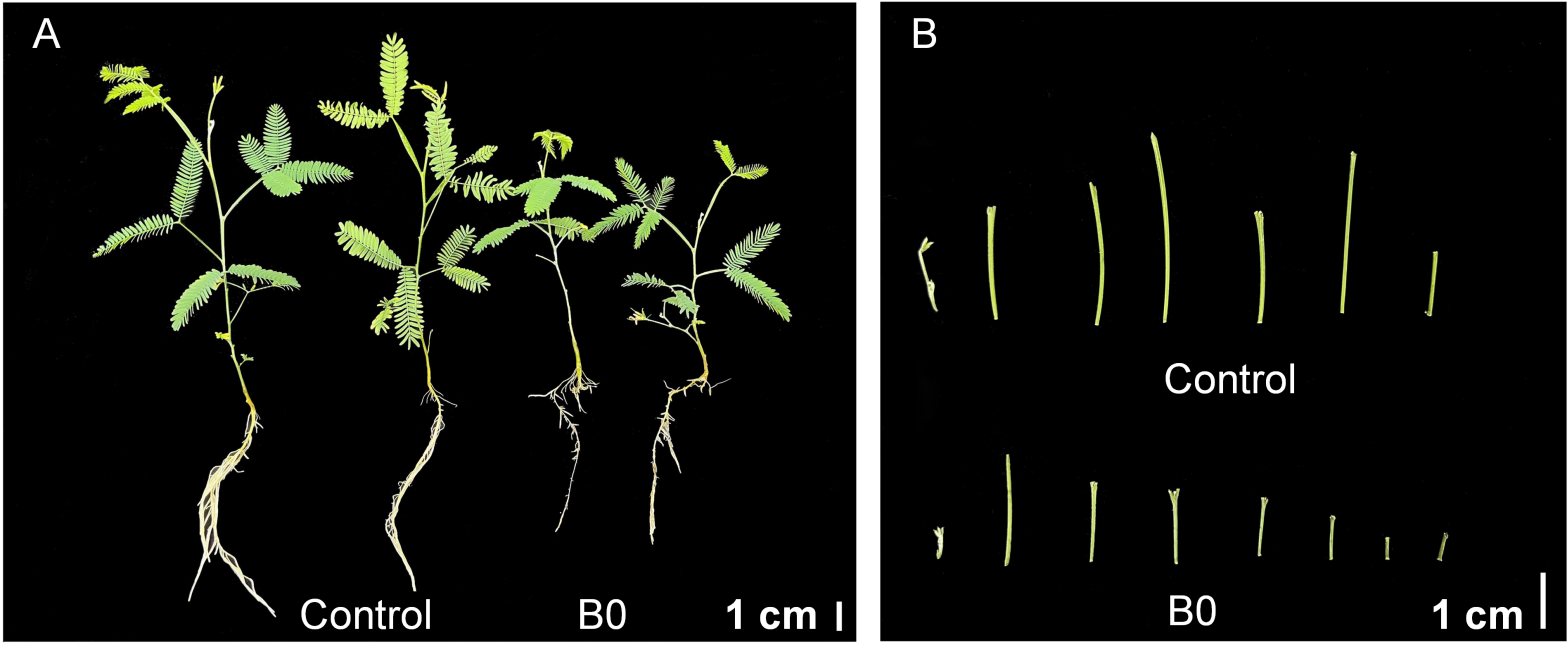
*A. melanoxylon* plants morphology under boron deficiency. **(A)** Phenotype; **(B)** Internodes morphology. Control (50 μM boric acid); B0 (0 μM boric acid). Scale bars of phenotype and internode morphology are 1 cm.

Under the boron deficiency (B0) condition, the phenotype of decreased plant height, shortened internodes, increased branches, and brown roots were observed, compared to the control (50 μM boric acid) (**Figures 1A, B**). The results of growth parameters shown in **Table 1** revealed that B0 treatment led to a 33.52% decrease in plant height, 27.76% decrease for the 1-st internode, 36.99% decrease for the 2-nd internode, and 42.59% decrease for the 3-rd internode, and a 118.33% increase in the branch number. Meanwhile, the primary root length and surface area were decreased by 32.08% and 24.89%, respectively. Interestingly, the root diameter increased by 41.37%. These results suggest that boron is essential for growth in *A. melanoxylon*.

**Table 1:** Growth parameters of *A. melanoxylon* under boron deficiency.

Treatment	plant height	branch number	internode length-1st	internode length-2nd	internode length-3rd	main root length	root surface area	root diameter
Control	9.87 ± 2.33	1.20 ± 0.40	1.70 ± 0.64	2.31 ± 0.71	2.24 ± 0.87	7.16 ± 1.19	13.03 ± 5.29	0.53 ± 0.13
B0	6.46 ± 1.68**	2.62 ± 0.97	1.23 ± 0.47	1.46 ± 0.67**	1.29 ± 0.76**	4.85 ± 1.55**	9.79 ± 3.25	0.75 ± 0.19**

Control (50 μM boric acid); B0 (0 μM boric acid). All data are the mean of three replicates collected over 60 days of treatment. Values are the mean ± standard deviations. The ** indicates significant differences by the Duncan test (P-value< 0.01).

### 3.2 Effect of boron deficiency on plant biomass and boron nutrient

**Figure 2:**
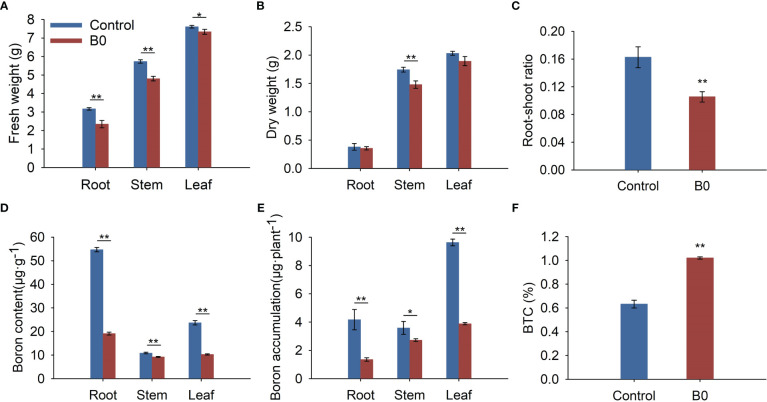
The biomass and boron nutrient of *A. melanoxylon* changes under boron deficiency. Control (50 μM boric acid); B0 (0 μM boric acid). **(A)** Fresh weight; **(B)** Dry weight; **(C)** Root-shoot ratio; **(D)** Boron content; **(E)** Boron accumulation; **(F)** BTC. All data are the mean of three replicates collected over 60 days of treatment. Data are the mean ± standard deviations. The * indicates significant differences at P-value< 0.05, and ** indicates significant differences at P-value< 0.01.

Under the B0 condition, the fresh weights, dry weights, and root-shoot ratio were decreased (**Figures 2A–C**). Meanwhile, boron deficiency led to a 65.07% reduction of boron content in roots, 15.22% in stems, and 56.68% in leaves (**Figure 2D**). Under the control condition, boron accumulation in roots was higher than in stems, but under the B0 condition, it was lower than in the stems (**Figure 2E**). Moreover, the BTCs of plants were 0.63 and 1.02 under control and B0 conditions, respectively (**Figure 2F**). These results suggest that *A. melanoxylon* preferentially transports boron from roots to shoots during long-term boron deficiency. The BEC coefficient of *A. melanoxylon* under the B0 condition is 90%, indicating that the soluble boron content is low and the boron utilization of the *A. melanoxylon* cultivar (SR17) is efficient.

### 3.3 Effects of boron deficiency on physiological indicators and endogenous hormone contents in stem

**Figure 3:**
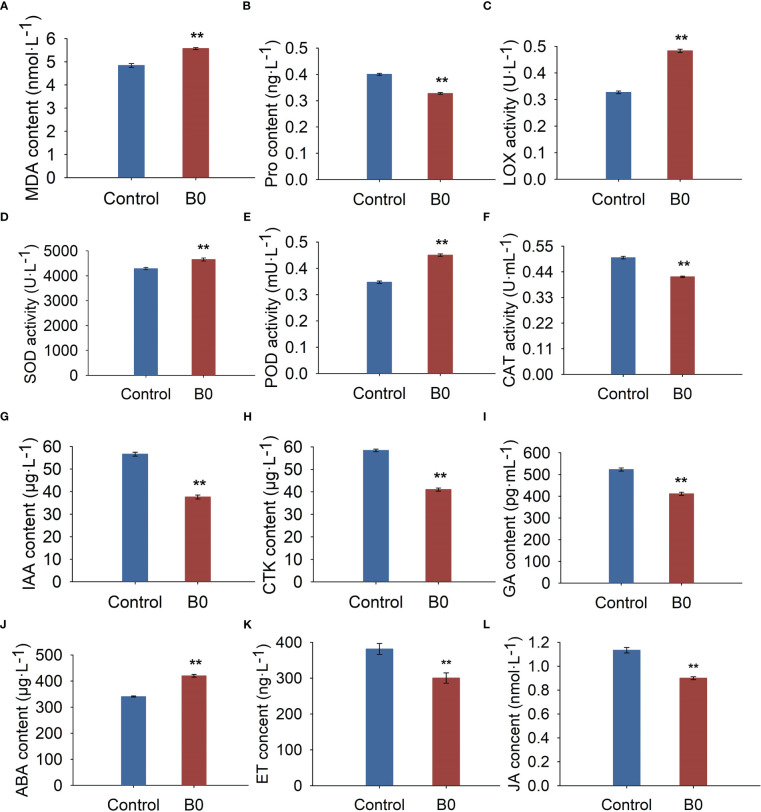
The physiology and hormone changes of *A. melanoxylon* stems under boron deficiency. Control (50 μM boric acid); B0 (0 μM boric acid). **(A)** MDA content; **(B)** Pro content; **(C)** LOX activity; **(D)** SOD activity; **(E)** POD activity; **(F)** CAT activity; **(G)** IAA content; **(H)** GA content; **(I)** CTK content; **(J)** ABA content; **(K)** ET content; **(L)** JA content. All data are the mean of three replicates collected over 60 days of treatment. Data are the mean ± standard deviations. The ** indicates significant differences at P-value< 0.01.

The experiments showed that boron deficiency led to an increase in MDA content (15.04%, **Figure 3A**), LOX activity (47.79%, **Figure 3C**), and a decrease in Pro content (18.14%, **Figure 3B**). These changes induced increases in SOD (8.48%, **Figure 3D**) and POD (29.75%, **Figure 3E**) activities and a decrease in CAT activity (16.42%, **Figure 3F**).

Compared to the control condition, the endogenous IAA, GA, CTK, ET, and JA contents were significantly reduced by 33.55%, 21.36%, 29.78%, 21.27%, and 20.63%, respectively (**Figures 3G–I, K, L**), while ABA content increased significantly by 23.44% under B0 condition (**Figure 3J**). These results indicate that *A. melanoxylon* altered its stem’s original endogenous hormone levels to adapt to boron deficiency.

### 3.4 Stem ultrastructure and cell wall components content

**Figure 4:**
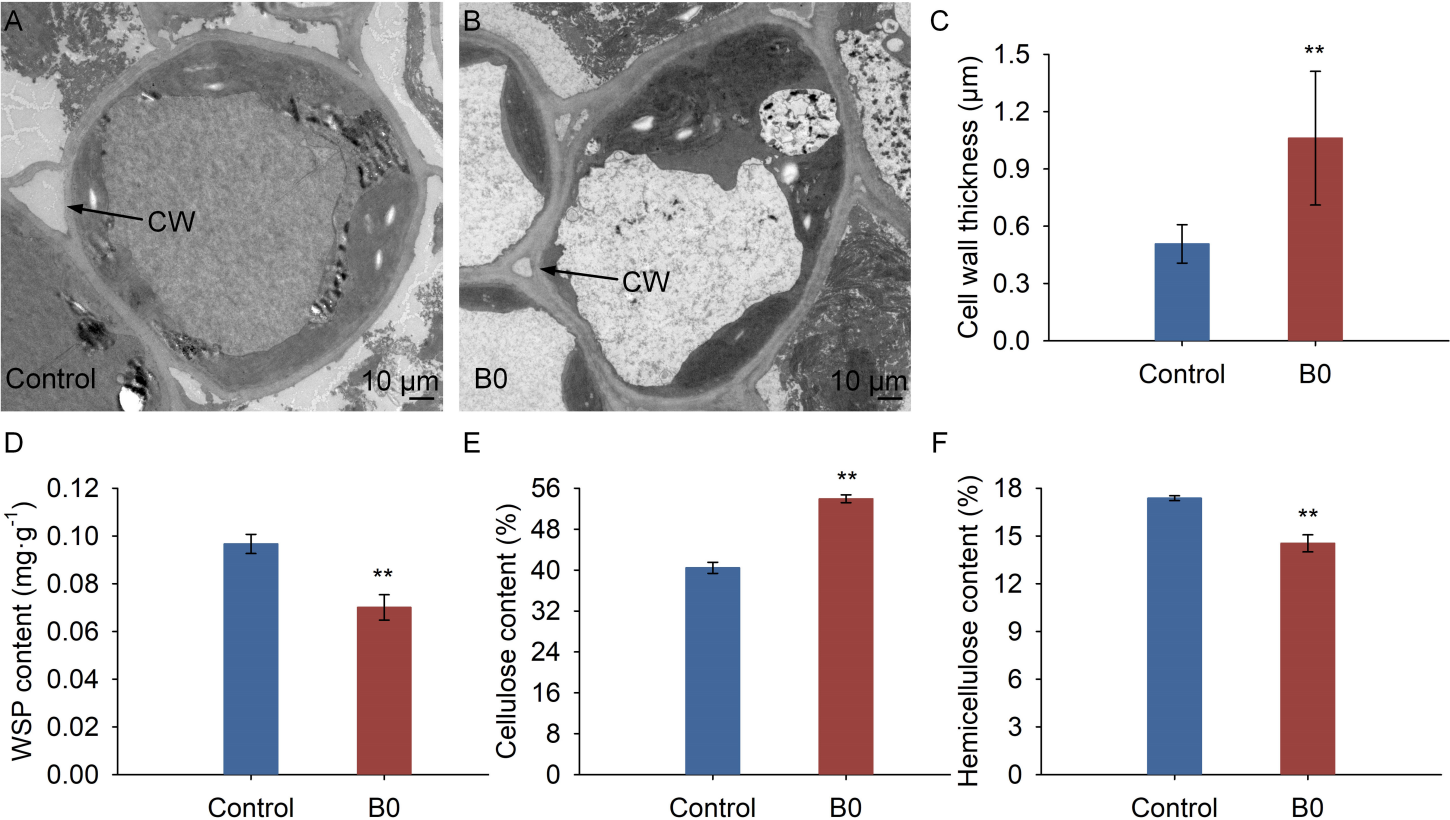
Structure and composition of cell wall under different boron conditions. Control (50 μM boric acid); B0 (0 μM boric acid). **(A)** Transmission electron microscope micrograph under control condition; **(B)** Transmission electron microscope micrograph under B0 condition; **(C)** Cell wall thickness; **(D)** WSP content means water soluble pectin content; **(E)** Cellulose content; **(F)** Hemicellulose content. All data are the mean of three replicates collected over 60 days of treatment. Data are the mean ± standard deviations. The ** indicates significant differences at P-value< 0.01.

TEM micrograph analysis showed that the cell wall of the stem thickened under boron deficiency (**Figure 4B**), while it remained regular under the control condition (**Figure 4A**). Results of ImageJ software showed that the cell wall thickness under the boron deficiency condition significantly increased by 2.08 times compared to the control condition (**Figure 4C**). Moreover, the WSP and hemicellulose contents were significantly decreased by 27.52%, and 16.33%, respectively, in comparison to the control condition (**Figures 4D, F**), whereas the cellulose content increased significantly by 33.35% under B0 condition (**Figure 4E**).

### 3.5 Transcriptome profiling of stem under boron deficiency

After removing low-quality and short reads, the number of clean reads ranged from 7.2 to 8.7 Gb, and the percentage of Q30 was more than 88.9% (**Supplementary File 2**). The 77.51% to 80.76% of unique reads can match the reference genome sequence (**Supplementary File 3**). We assessed the similarities and differences among samples using Pearson correlation analysis and PCA. The Pearson correlation coefficient (R^2^) between samples was higher than 0.87, and the clustering was obvious (**Supplementary Figures 1A, B**), indicating that the transcriptome data are reliable for subsequent analysis.

The gene expression levels were measured according to FPKM, with all samples restricted to the range 4 ≥ log_10_ (FPKM) ≥ -2 (**Supplementary Figure 2A**). Transcriptome analysis identified a total of 5012 DEGs, of which 2348 genes were upregulated and 2264 genes were down-regulated (**Supplementary Figure 2B**). A volcano plot was utilized to display the FC values in gene expression (**Supplementary Figure 2C**). Furthermore, to better understand the overall variation in DEG expression, a heatmap was built to visualize the expression patterns of all DEGs. The DEGs were classified into 3 clusters based on their expression patterns (**Supplementary Figure 2D**). These results indicate that there is a considerable change in the transcription levels of many genes in *A. melanoxylon* stem under boron deficiency.

### 3.6 Real-time qPCR validation

To verify the authenticity and reproducibility of the transcriptomic data, we selected 15 DEGs and designed specific primers for qRT-PCR. The relative expression of the selected genes was compared with the results of RNA-seq analysis. The results showed that the 15 genes differed slightly from the sequencing data expression. Still, the overall expression trend was identical (**Supplementary Figure 3**), confirming the reliability of this study’s transcriptome sequencing results.

### 3.7 GO and KEGG pathways analysis of DEGs

**Figure 5:**
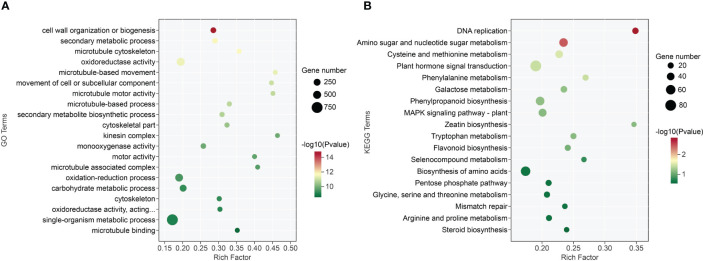
GO function and KEGG enrichment pathway analysis of *A. melanoxylon* stems under boron deficiency. **(A)** GO function analysis. “Oxidoreductase activity, acting…” means oxidoreductase activity, acting on paired donors, with incorporation or reduction of molecular oxygen, NAD(P)H as one donor, and incorporation of one atom of oxygen. **(B)** KEGG enrichment pathway analysis.

The 406 differentially expressed GO terms (P-value< 0.05) were identified through GO enrichment analysis. Specifically, the biological process (BP), molecular functions (MF), and cell component (CC) included 224, 125, and 57 terms, respectively (**Supplementary File 4**). The cell wall organization or biogenesis, oxidoreductase activity, and microtubule cytoskeleton were the most significantly enriched GO terms in BP, MF, and CC ontology, respectively. Besides these categories, DEGs were functionally related to lignin metabolism, secondary metabolism, and DNA replication processes (**Figure 5A**; **Supplementary File 4**).

To identify DEGs enriched in various metabolic pathways, the KEGG database was utilized, with a P-value< 0.05 as the screening threshold. The DEGs were categorized into 18 functional categories, including 2 environmental information processing, 2 genetic information processing, and 14 metabolism pathways (**Supplementary File 5**). The plant hormone signal transduction, biosynthesis of amino acids, and phenylpropanoid biosynthesis were identified as the most abundant KEGG pathways (**Figure 5B**).

### 3.8 Response of cell wall organization or biogenesis-related DEGs under boron deficiency

**Figure 6:**
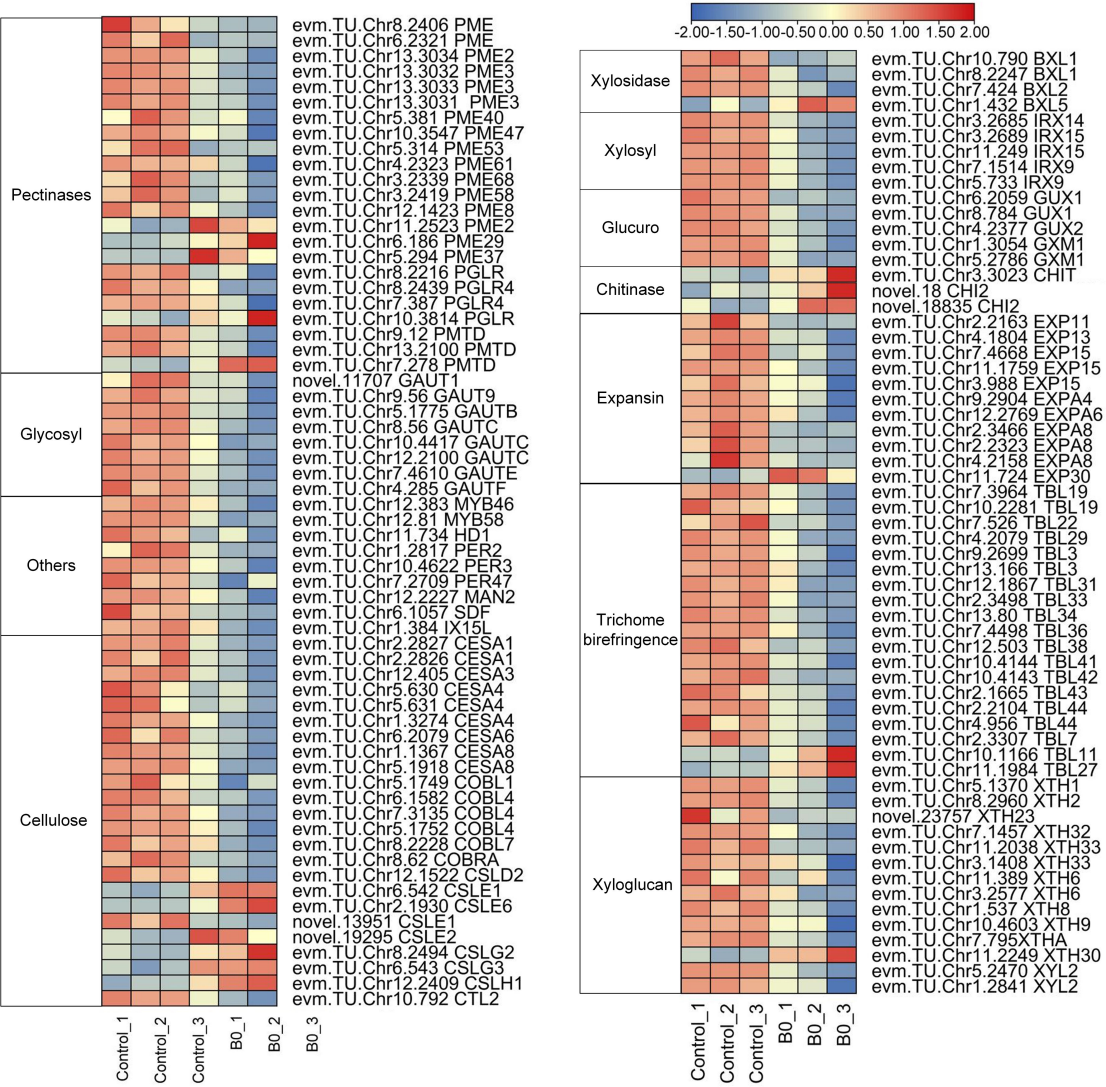
Expression of DEGs for cell wall organization or biogenesis under boron deficiency. The heatmaps show log_2_FPKM values of the DEGs. Red indicates up-regulation and blue indicates down-regulation.

DEGs related to cell wall organization or biogenesis were examined to further investigate the effects of boron deficiency on cell wall structure and composition. 127 DEGs encoding proteins or enzymes were involved in cell wall metabolism, of which 106 were down-regulated and 21 were upregulated under boron deficiency (**Figure 6**; **Supplementary File 6**). 28 DEGs encoding pectinaceous components or pectin-modifying enzymes, such as *pectinesterase* (*PMEs*), *polygalacturonase* (*PGLs*), *pectin acetylesterase* (*PAEs*) and *galacturonosyltransferase* (*GAUT*) were identified, of which 5 were upregulated (*evm.TU.Chr11.2523 PME2*, *evm.TU.Chr6.186 PME29*, *evm.TU.Chr5.294 PME37*, *evm.TU.Chr10.3814 PGLR*, and *evm.TU.Chr7.278 PMTD*) and 23 down-regulated (**Supplementary File 6**). 44 DEGs encoded structural components of cell walls such as *cellulose*, *glucans*, *xylans*, and *galacturonans*. 44 DEGs encoded proteins such as *expansins* (*EXPs*), *trichome birefringence-like* (*TBLs*), and *xyloglucan endotransglucosylase/hydrolase* (*XTHs*), which are required for cell wall loosening during growth. In addition, 3 TFs (*evm.TU.Chr12.383 MYB46*, *evm.TU.Chr12.81 MYB58*, and *evm.TU.Chr11.734 HD1*) were downregulation under boron deficiency.

### 3.9 Transcription factors regulate cell wall-related genes under boron deficiency

A total of 314 DEGs encoding TFs (**Supplementary File 7**) were found in the transcriptome database, of which 171 were upregulated and 143 were downregulated. Among these, *MYBs* were the most abundant family (56), followed by *AP2-EREBPs* (43), *bHLHs* (26), *NACs* (25), and *WRKYs* (18) in turn.

**Figure 7:**
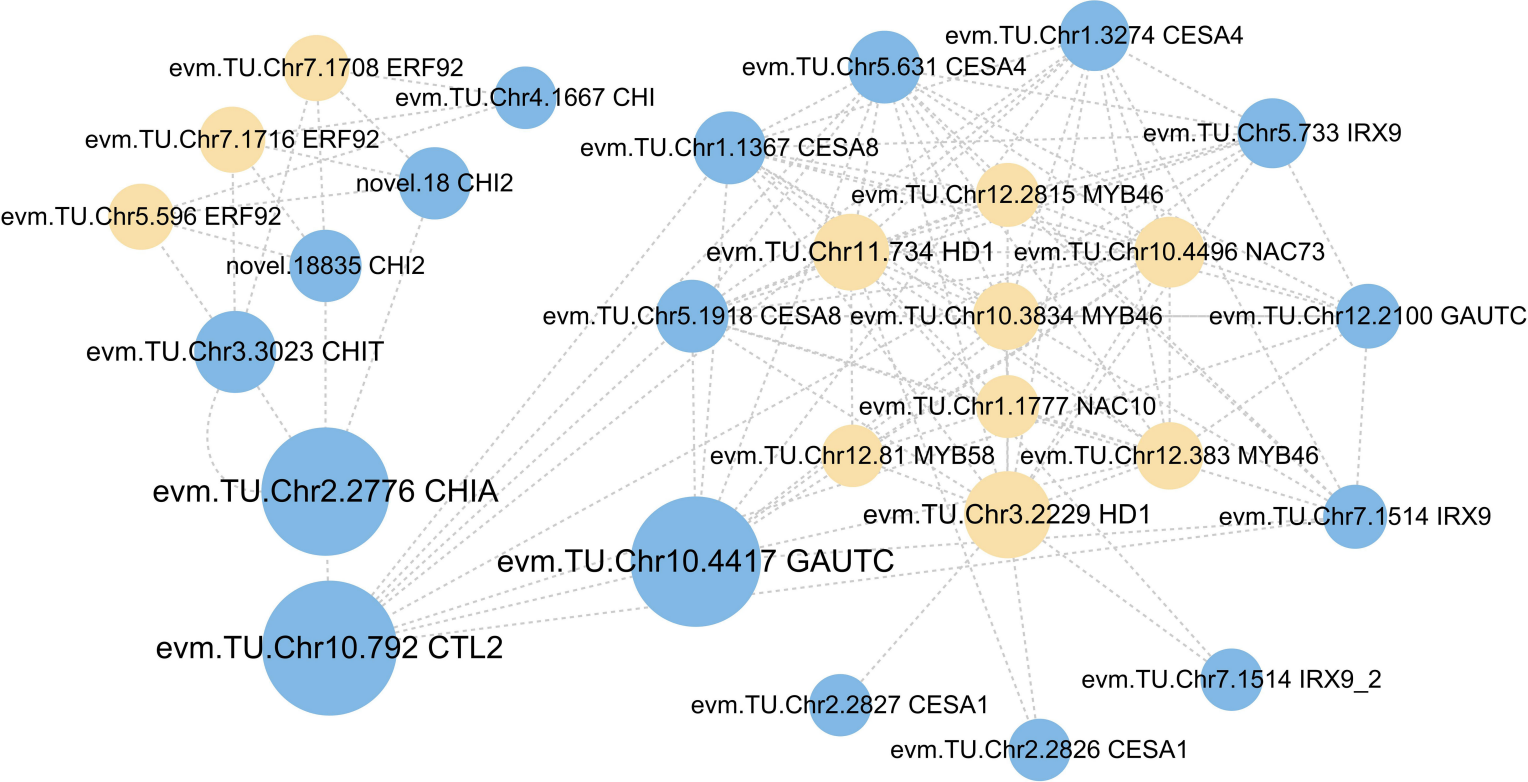
The network of transcription factors with cell wall organization or biogenesis-related genes. Cycle nodes represent genes, yellow nodes represent the transcription factors, while blue nodes represent cell wall organization or biogenesis-related genes. The size of node represents the power of the interrelation among the nodes by degree value.

The interaction network of TFs and cell wall organization or biogenesis-related genes was constructed to reveal the potential regulatory mechanisms in *A. melanoxylon* stem under boron deficiency. As shown in **Figure 7**, cell wall organization or biogenesis-related genes, such as *GAUTCs*, *CESAs*, and *IRXs*, were found to interact with TFs, containing 2 *HD1s*, 1 *NAC73*, and 3 *MYB46s*. Moreover, *NAC10* and *MYB58* served as master switches in charge of the transcriptional regulation of the cell wall network. It is noteworthy that 2 *HD1s* interacted not only with *MYBs* and *NACs* but also with several *CESAs* and *IRXs*. In addition, 3 *ERF92s*, which are part of the ET signaling pathway, interact with 4 *CHIs*.

### 3.10 Response of plant hormone-related DEGs under boron deficiency

**Figure 8:**
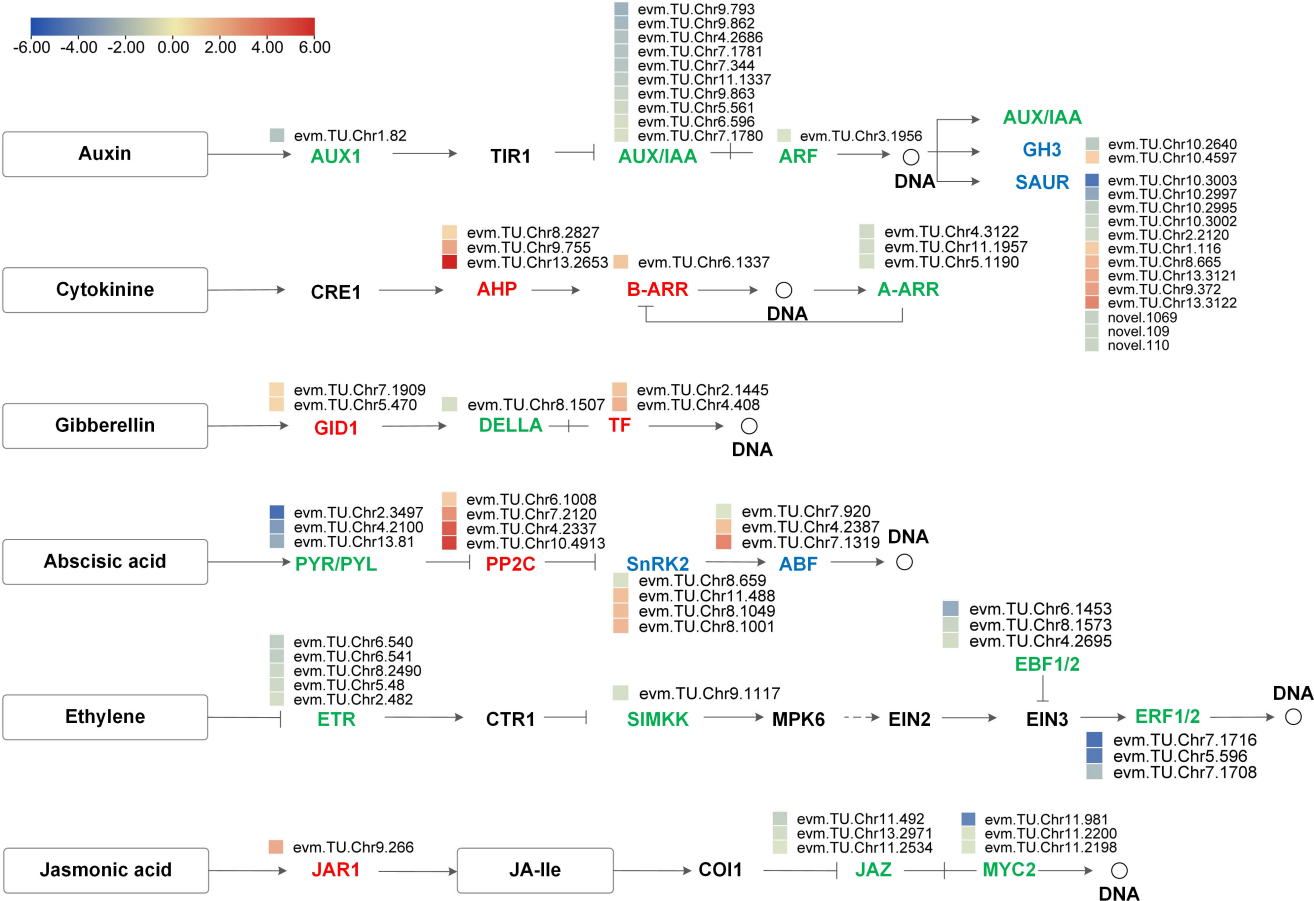
Process map of plant hormone signal transduction pathway for auxin, cytokinin, gibberellin, abscisic acid, ethylene and jasmonic acid. The heatmaps show log_2_FC values of the DEGs. Red indicates up-regulation and blue indicates down-regulation.

Based on the DEGs’ enrichment results, there were 81 DEGs involved in the plant hormone signal transduction pathways, of which 30 were upregulated and 51 were downregulated (**Supplementary File 8**). The number of DEGs involved in the IAA signaling pathway was the most, followed by ABA and ET signaling pathways. We further analyzed the expression pattern of DEGs in IAA, CTK, GA, ABA, ET, and JA pathways and visualized them with process maps. As shown in **Figure 8**, all 11 DEGs in the ET pathway were down-regulated. Hormone signal receptors (*GID1s*, *PYR/PYLs*, and *ETRs*) and response regulator (*AUX1s*, *AUX/IAAs*, *ARFs*, *A-ARRs*, *DELLAs*, and *ERFs*) genes were also all down-regulated. In the JA signal transduction pathway, boron deficiency upregulated *JAR1*, leading to the downregulation of downstream protein *JAZs* and TF *MYC2s.* These results indicate that the gene expression changes associated with plant hormone signal transduction are likely to be implicated in the growth regulation of *A. melanoxylon* stem under boron deficiency.

To clarify the complex interaction network between hormones, we drew an interaction network diagram between DEGs (**Supplementary Figure 4**). *ABF* (*evm.TU.Chr.4.2387*) belonging to the ABA signal transduction pathway, interacted with the *AUX/IAA* genes (*evm.TU.Chr5.561* and *evm.TU.Chr9.973*) involved in the IAA signaling pathway. The *AHPs* (*evm.TU.Chr9.755*, *evm.TU.Chr8.2827*, and *evm.TU.Chr13.2653*) belonging to the CTK signal transduction pathway, interacted with *ETR92s* (*evm.TU.Chr6.540*, *evm.TU.Chr8.2490* and *evm.TU.Chr6.541*) involved in the ET signal transduction pathway.

## 4 Discussion

### 4.1 Boron deficiency inhibits the growth and development of *A. melanoxylon*

The present study showed that *A. melanoxylon* presented various symptoms under boron deficiency, such as dwarf plants, increased branches, shortened internodes, and notably shorter and thicker roots (**Figure 1**). The morphological changes observed in plants may be the end phenotypic results of altering cell wall integrity and hormone homeostasis-related pathways. For instance, Yin et al. (2022) found that severe morphological changes induced by boron deficiency may be caused by damaging the cell wall integrity in *N. Cadamba*. Chen et al. (2022) reported that boron deficiency induced jasmonate signaling and remodeling of cell wall metabolism in pea (*Pisum sativum*) shoots, which was the reason for changes in shoot growth and architecture. In our study, long-term boron deficiency significantly altered the cell wall structure, composition, and endogenous hormone levels, resulting in morphological changes in *A. melanoxylon* stem. In addition, antioxidant enzyme (SOD, POD, CAT, and LOX) activities, antioxidant and oxidative stress indicators were also significantly changed in response to boron deficiency in *A. melanoxylon* stem (**Figure 3**). The reason may be that cells altered their osmotic potential and activated the antioxidant mechanism to protect cell membranes and maintain oxidative homeostasis under boron deficiency stress (Zhu, 2016; Zhu et al., 2020).

### 4.2 Boron deficiency affects cell wall organization or biogenesis in *A. melanoxylon* stem

The growth inhibition of apical meristems was one of the early responses to boron deficiency, which is attributable to the loss of cell wall plasticity (Dell and Huang, 1997; Chen et al., 2023). Boron deficiency disrupts the structural arrangement of the cell wall, which in turn affects cell function and cell wall components (Wang et al., 2015; Brdar-Jokanović, 2020). Boron crosslinked with RG-II in the cell wall and was closely associated with the biosynthesis of pectin, cellulose, and lignin (Hu and Brown, 1994; Wu et al., 2017; Yan et al., 2022). Boron deficiency caused the cell wall of *A. melanoxylon* stem to thicken, with a concomitant decrease in the hemicellulose and WSP contents (**Figure 4**). At the molecular level, boron deficiency caused a significant reduction in the expression of the great majority of genes involved in cell wall organization or biogenesis pathway in *A. melanoxylon* stem, as shown in **Figure 5** and **Supplementary File 3**. A previous study has also reported that boron deficiency downregulates the expression of several cell wall-related genes in *Arabidopsis* roots (Camacho-Cristóbal et al., 2008). These results suggest that boron deficiency affects the structure and composition of the cell wall and is also involved in the expression of cell wall-related genes. Furthermore, the expression patterns of cell wall-related genes have been demonstrated to be connected with plant morphology. For example, overexpressing *PmCESA2* in poplar increased secondary cell wall thickness and xylem width, leading to higher cellulose and lignin content, and improved biomass production (Maleki et al., 2020). In *Arabidopsis*, overexpressing *AtEXPA4* enhanced primary root elongation, while knocking out *AtEXPA4* slowed down primary root growth (Liu et al., 2021). However, the functions of these genes in *A. melanoxylon* have rarely been reported. To gain a better understanding of the effects of boron deficiency on *A. melanoxylon* stem, further investigations are needed to identify the genes that are either activated or repressed by boron, and how they affect the cell wall structure and composition.

### 4.3 Boron deficiency induced interactions of cell wall and hormone-related genes in *A. melanoxylon* stem

Cell wall integrity is an essential foundation of plant growth and development. Previous studies have indicated that boron deficiency can lead to changes in cell wall integrity and endogenous phytohormone balance. For instance, in *Arabidopsis*, Camacho-Cristóbal et al. (2015) found that boron deficiency damages cell wall integrity and activates ethylene, auxin, and ROS signaling pathways, thus causing a rapid reduction in root elongation. Additionally, Chen et al. (2022) proposed that boron deficiency disorders cell wall structure, thereby triggering the activation of JA signaling and subsequent compensatory changes in cell wall metabolism. Consequently, it can be inferred that there is an intricate interaction between cell walls and hormonal signaling mechanisms in response to boron deficiency. This hypothesis has also been validated by studies conducted on other plant species. Specifically, mutations in *AtCESAs* inhibit cellulose biosynthesis, leading to the redistribution of the auxin efflux carrier *AtPIN1* in the shoot apical meristem, thereby significantly affecting shoot apical meristem development (Sampathkumar et al., 2019). Our research found that 3 ET response factors (*ERF92s*) interact with the 4 class II chitinases (*CHIs*) (**Figure 7**). Previous research has shown that *CHI* participates in the catabolic process of cell wall macromolecules (De Andrade Silva et al., 2020). Under boron deficiency, *ERF92s* were downregulated, while *CHIs* were upregulated in *A. melanoxylon* stem (**Supplementary Files 6**, **8**). This suggests that the upregulation of *CHIs* expression may enhance the disassembly of cell wall macromolecules, thereby impeding the transduction of ET signaling.

Previous studies have reported the existence of crosstalk among plant hormones under abiotic stress, forming complex signal transduction networks (Waadt et al., 2022). Additionally, it has been found that boron-related morphological and physiological disorders are associated with the production and signal transduction of plant hormones (Eggert and von Wirén, 2017; Chen et al., 2023). For example, in *Arabidopsis* seedlings, the crosstalk between CTKs, ETs, and IAAs functions acts as a signal in response to boron deficiency, regulating root cell elongation and boron transport (Herrera-Rodríguez et al., 2022). The interaction among IAAs, CTKs, and GAs also impacts plant stem elongation (Santner et al., 2009; Hedden and Thomas, 2012; Singh and Roychoudhury, 2022). In our study, we observed that the *ERF92s* belonging to the ET signaling pathway interacted with the *AHPs* belonging to the CTK signal transduction pathway (**Figure S4**). This indicates that CTK affects the cell wall of *A. melanoxylon* plants in a boron deficiency environment through its interaction with ET. Further research is needed to investigate the relationship between other hormonal signals and plant development and cell wall integrity under boron deficiency stress.

### 4.4 Boron deficiency induces TFs that regulate cell wall-related genes in *A. melanoxylon* stem

Previous studies have reported that *MYBs* and *NACs* are master transcriptional switches of the secondary cell wall. They regulate the expression of genes involved in the biosynthesis of cellulose, xylan, glucomannan, and lignin (Zhong and Ye, 2014). *AtMYB46* directly regulates the expression of secondary wall-associated *CESAs* in *Arabidopsis* (Kim et al., 2013). In *Thellungiella halophila*, the co-overexpression of *TsHD1* and *TsNAC1* significantly inhibits plant growth by restraining cell expansion (Liu et al., 2019). In our study, network analysis reveals that *MYBs* (*evm.TU.Chr12.2815*, *evm.TU.Chr12.81*, *evm.TU.Chr10.3834*, and *evm.TU.Chr12.383*), *NACs* (*evm.TU.Chr1.1777* and *evm.TU.Chr10.4496*) and *HD1s* (*evm.TU.Chr11.734* and *evm.TU.Chr3.2229*) regulate numerous DEGs involved in cell wall organization or biogenesis (**Figure 7**). Our research also found that these *HD1s*, *NACs*, and *MYBs* exhibit significant down-regulation under boron deficiency (**Supplementary File 7**). This suggests that long-term boron deficiency inhibits TFs-mediated processes in cell wall organization or biosynthesis, ultimately retarding the development of *A. melanoxylon* stem.

**Figure 9:**
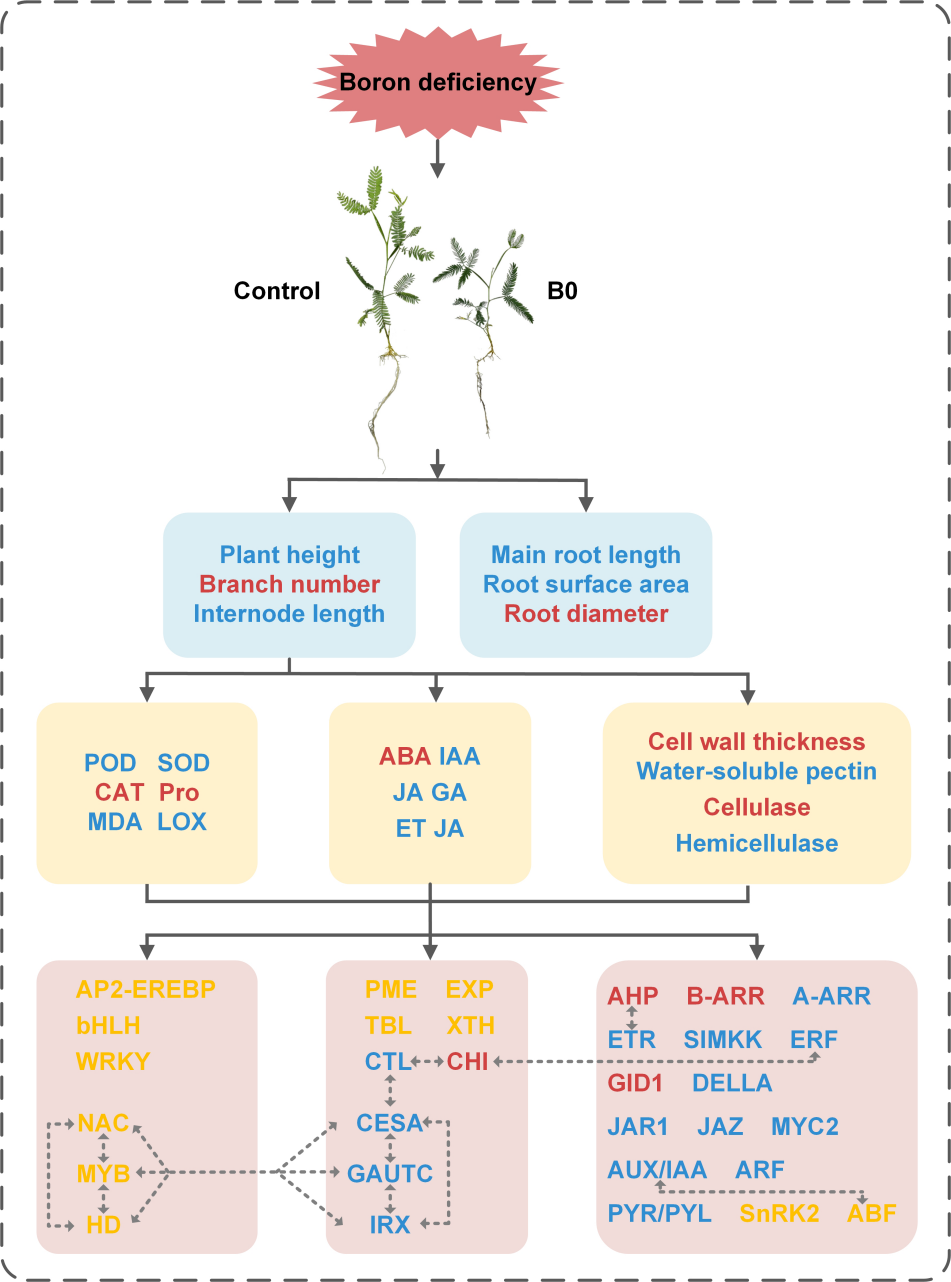
Schematic model of changes in the physiological and molecular mechanisms of *A. melanoxylon* stem under boron deficiency. The dashed line with arrows indicate speculative rules inferred by RNA-seq enrichment of DEGs. Red words represent the up-regulation of substances or genes, blue word represent down-regulation, and yellow words represent both up-regulation and down-regulation of genes.

Finally, we built a schematic model to visualize the process of *A. melanoxylon* in response to boron deficiency, as shown in **Figure 9**. Boron deficiency causes changes in boron nutrition, leading to oxidative stress and alterations in cell wall structure and composition. Hormone biosynthesis, transport, and signal transduction are also disturbed. In addition, TFs and plant hormone signal transduction-related genes impact cell wall organization or biosynthesis, suggesting that the response of plants to boron deficiency is a complex regulatory process. Taken together, the inhibitory effects of boron deficiency stress on the growth and development of *A. melanoxylon* stem are attributed to changes in cell wall structure and composition and transcriptional regulation. This study provides a theoretical basis for further understanding the response mechanism of woody plants to boron deficiency stress.

## Data availability statement

The datasets presented in this study can be found in online repositories. The names of the repository/repositories and accession number(s) can be found in the article/**Supplementary Material**.

## Author contributions

ZC: Investigation, Data curation, Formal Analysis, Writing – original draft. XB: Data curation, Formal Analysis, Investigation, Writing – original draft. BZ: Data curation, Funding acquisition, Investigation, Methodology, Writing – review & editing. CF: Writing – review & editing. XL: Methodology, Writing – review & editing. BH: Conceptualization, Supervision, Methodology, Validation, Data curation, Writing – review & editing.

## Supplementary material

Supplementary Figure 1 Pearson correlation and principal component analysis between samples.

Supplementary Figure 2 The differentially expressed genes (DEGs) in *A. melanoxylon* stem.

Supplementary Figure 3 qRT-PCR validation of 15 DEGs. Data are the mean ± standard deviations (n=3).

Supplementary Figure 4 Correlation network diagram of plant hormone signal transduction pathway (KEGG: ko04075).
