# Supplementary figures and images for: Physiological and molecular mechanisms of Acacia melanoxylon stem in response to boron deficiency
Source: Front Plant Sci. 2023 Oct 27;14:1268835. doi: 10.3389/fpls.2023.1268835 (PMC10641760; doi:10.3389/fpls.2023.1268835)

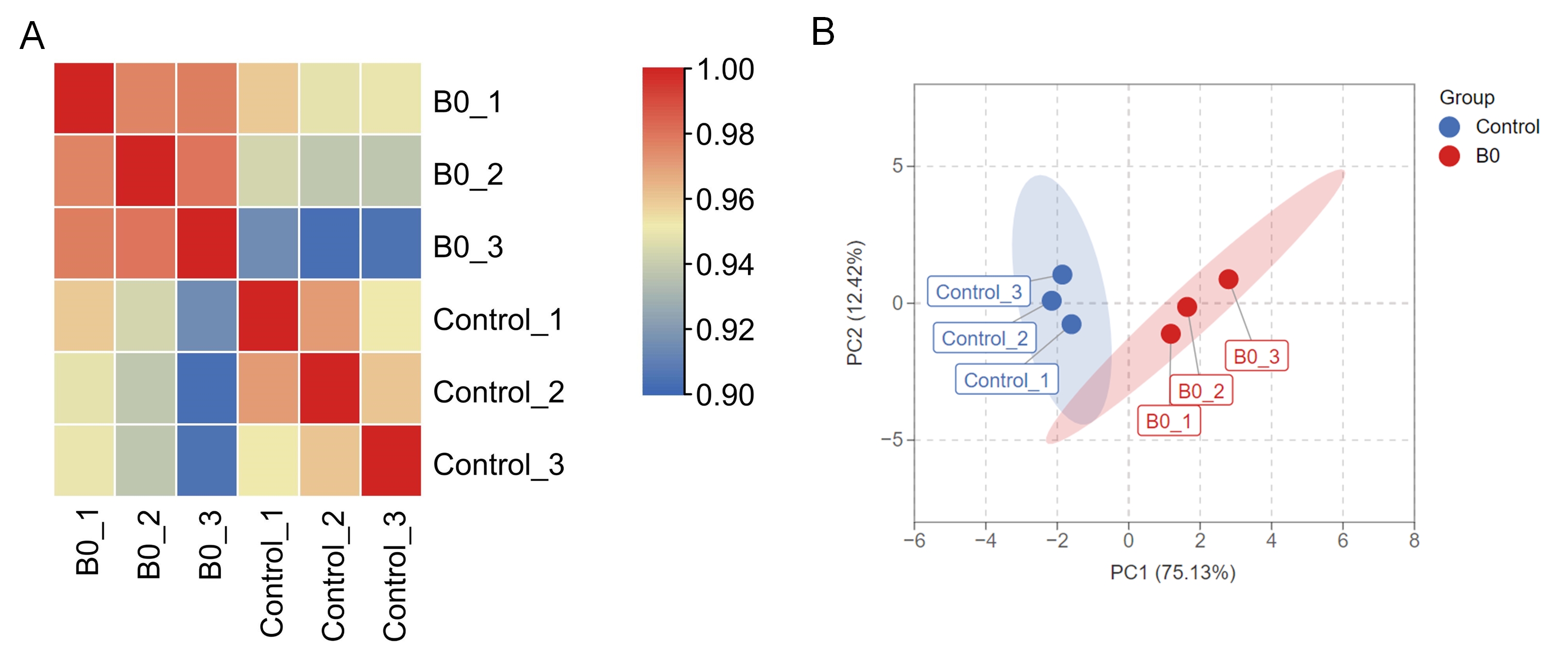

Supplement: Supplementary Figure 1 — Pearson correlation and principal component analysis between samples. [file Image_1.jpeg]

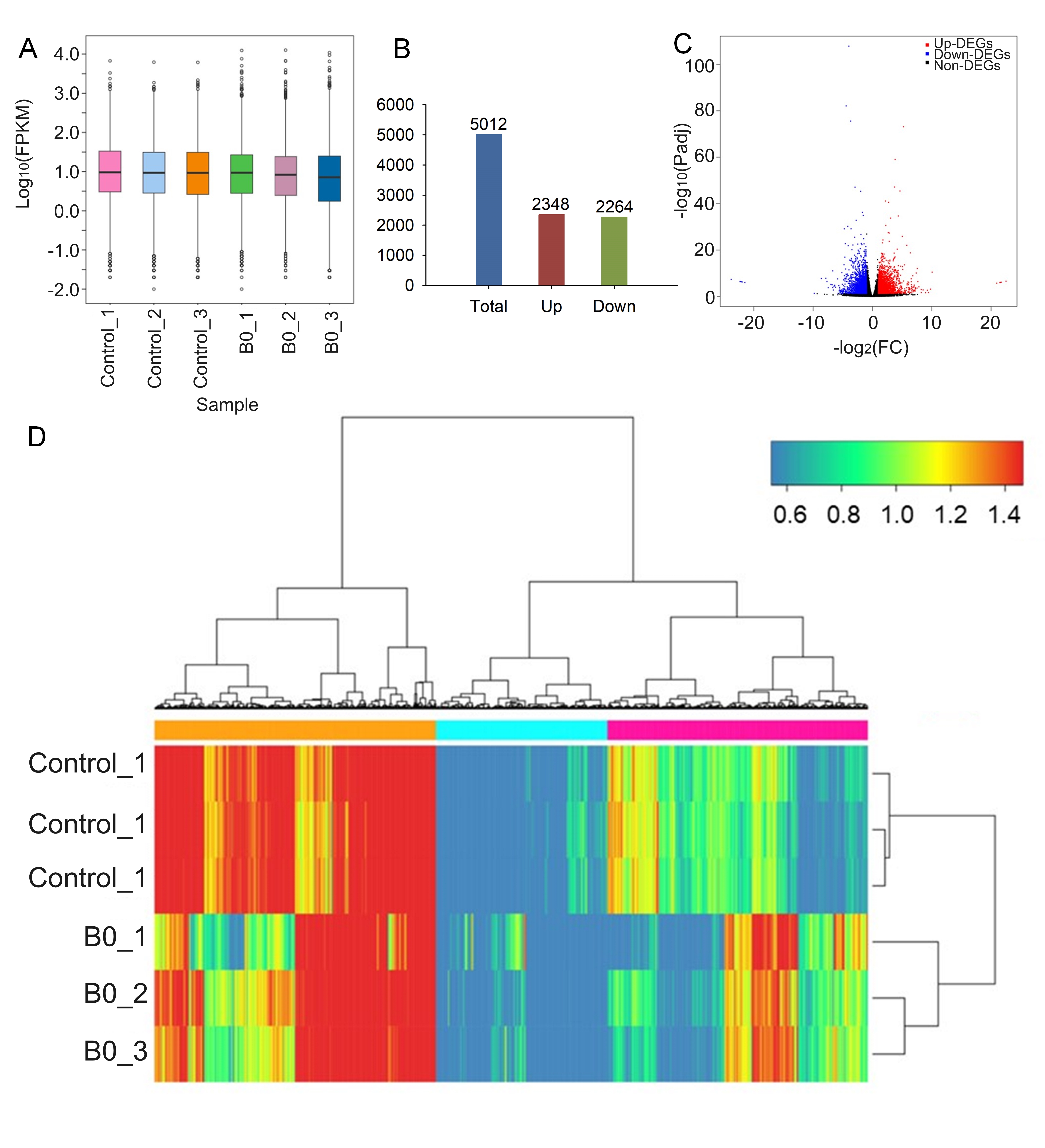

Supplement: Supplementary Figure 2 — The differentially expressed genes (DEGs) in A. melanoxylon stem. [file Image_2.jpeg]

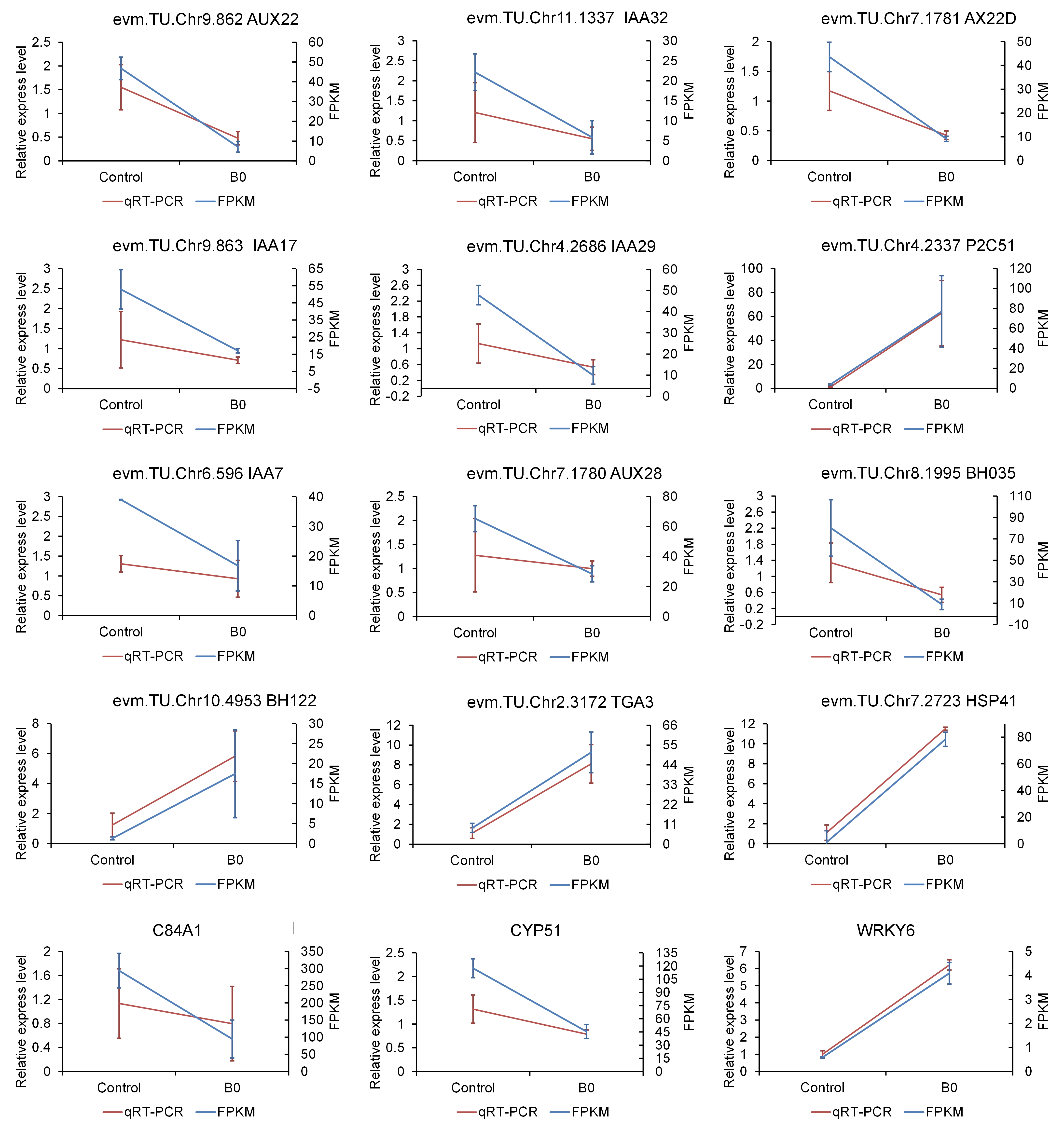

Supplement: Supplementary Figure 3 — qRT-PCR validation of 15 DEGs. Data are the mean ± standard deviations (n=3). [file Image_3.jpeg]

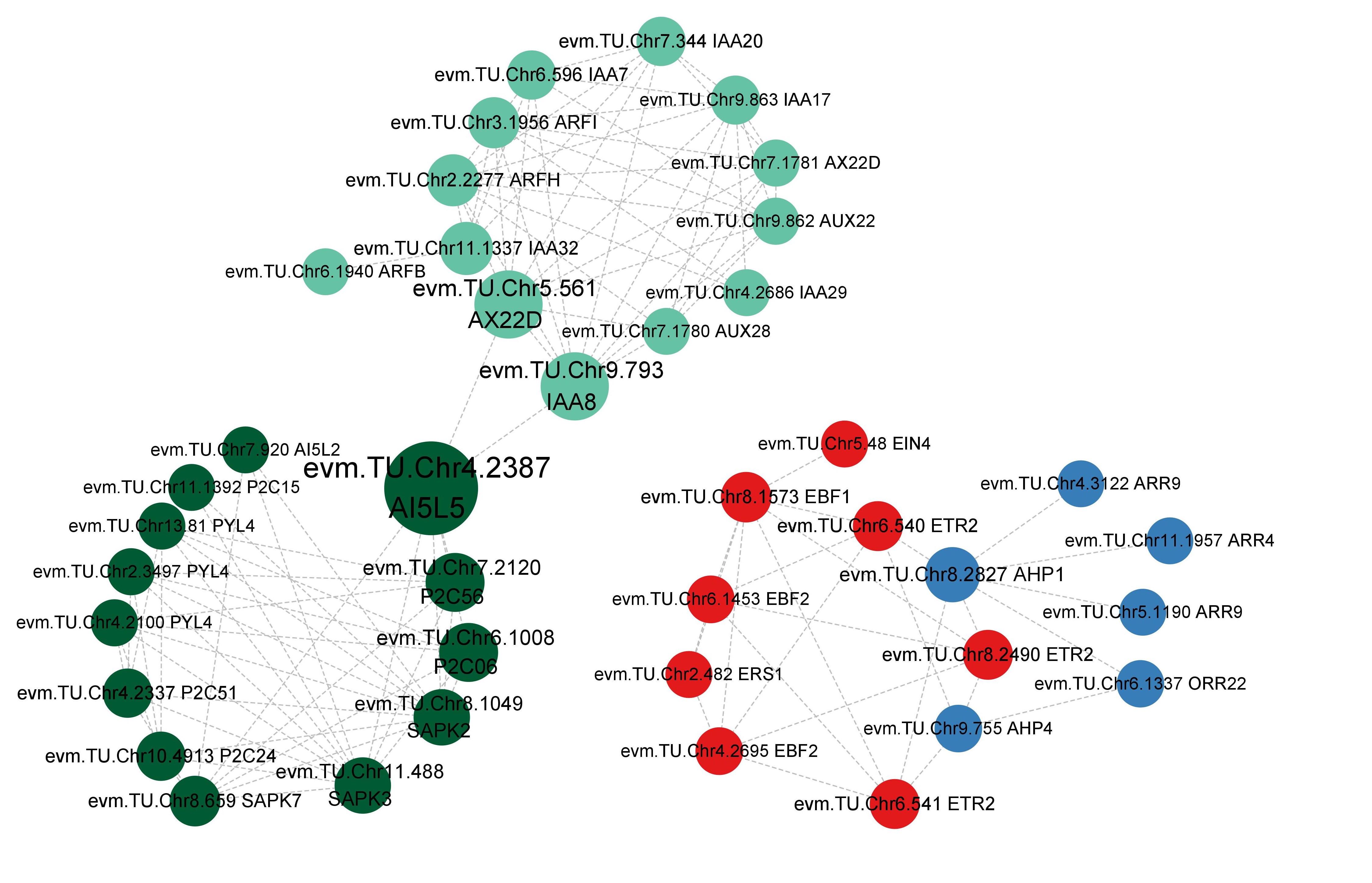

Supplement: Supplementary Figure 4 — Correlation network diagram of plant hormone signal transduction pathway (KEGG: ko04075). [file Image_4.jpeg]
